# A Novel Rolling Circle Amplification-Based Detection of SARS-CoV-2 with Multi-Region Padlock Hybridization

**DOI:** 10.3390/diagnostics12092252

**Published:** 2022-09-18

**Authors:** Rajesh Kumari, Ji Won Lim, Matthew Ryan Sullivan, Rachel Malampy, Connor Baush, Irina Smolina, Howard Robin, Vadim V. Demidov, Giovanni Stefano Ugolini, Jared R. Auclair, Tania Konry

**Affiliations:** 1 Department of Pharmaceutical Sciences, Northeastern University, 360 Huntington Avenue, Boston, MA 02115, USA; 2 Life Science Testing Center, Northeastern University, Burlington, MA 01803, USA; 3 Genemend, LLC, Yarmouth, MA 02180, USA; 4 LJ Pathology Consultants, La Jolla, CA 92037, USA; 5 Biotechnology & Pharmaceuticals Group, Global Prior Art, Inc., Boston, MA 02109, USA

**Keywords:** severe acute respiratory syndrome coronavirus (SARS-CoV-2), rolling circle amplification (RCA), multi-region padlock

## Abstract

SARS-CoV-2 has remained a global health burden, primarily due to the continuous evolution of different mutant strains. These mutations present challenges to the detection of the virus, as the target genes of qPCR, the standard diagnostic method, may possess sequence alterations. In this study, we develop an isothermal one-step detection method using rolling circle amplification (RCA) for SARS-CoV-2. This novel strategy utilizes a multi-padlock (MP-RCA) approach to detect viral-RNA via a simplified procedure with the reliable detection of mutated strains over other procedures. We designed 40 padlock-based probes to target different sequences across the SARS-CoV-2 genome. We established an optimal one-step isothermal reaction protocol utilizing a fluorescent output detected via a plate reader to test a variety of padlock combinations. This method was tested on RNA samples collected from nasal swabs and validated via PCR. S-gene target failure (SGTF)-mutated strains of SARS-CoV-2 were included. We demonstrated that the sensitivity of our assay was linearly proportional to the number of padlock probes used. With the 40-padlock combination the MP-RCA assay was able to correctly detect 45 out 55 positive samples (81.8% efficiency). This included 10 samples with SGTF mutations which we were able to detect as positive with 100% efficiency. We found that the MP-RCA approach improves the sensitivity of the MP-RCA assay, and critically, allows for the detection of SARS-CoV-2 variants with SGTF. Our method offers the simplicity of the reaction and requires basic equipment compared to standard qPCR. This method provides an alternative approach to overcome the challenges of detecting SARS-CoV-2 and other rapidly mutating viruses.

## 1. Introduction

The current ongoing coronavirus disease (COVID-19) caused by infection of severe acute respiratory syndrome coronavirus 2 (SARS-CoV-2, also known as HCOV-19) remains a potential threat to global public health. According to the World Health Organization (WHO) and the Center for Disease Control (CDC) in the United States, the gold-standard protocol for diagnosing SARS-CoV-2 infection is real-time fluorescence quantitative PCR (qPCR) targeting certain coding regions, including S, N, and ORF protein coding genes of SARS-CoV-2. While qPCR is a highly sensitive and accurate diagnostic tool, qPCR possesses some major limitations, including a relatively long reaction time, lower sensitivity for early-stage infection, and vulnerability in detecting the newly emerging mutations and variants, including new sub-variants of the Omicron SARS-CoV-2 strains [1,2,3]. 

SARS-CoV-2 is a genetically heterogenous virus, and identifying new emerging variants in clinical samples has become a major challenge of this pandemic. WHO has officially listed the Alpha, Beta, Gamma, Delta, Omicron, and other variants worldwide, with some variants producing higher transmissibility or infectivity even in vaccinated individuals [4,5,6,7]. Recently, the Omicron subvariant BA.2 has presented concerns as its high number of genomic mutations make it difficult to identify this strain correctly through PCR [8]. If the mutation occurs in region(s) of the viral genome targeted by the qPCR primers, new primers must be designed, requiring another round of the verification process enforced by governmental authorities. A wider fraction of the genome must be targeted simultaneously, including genetically stable and variable regions, to ensure the proper detection of all variants through a nucleic acid amplification method. 

To alternate and improve the current conventional qPCR-based diagnostic assays, many researchers have suggested alternative nucleic acid amplifying diagnostic methods including droplet digital PCR (ddPCR), loop-mediated isothermal amplification (LAMP), rolling circle amplification (RCA), DNA strand displacement amplification (SDA), and other methods. Among those efforts, the isothermal nucleic acid amplification methods including LAMP and RCA have been explored for the rapid detection of not only DNA but also viral RNA [9,10,11,12,13]. Typically, however, these techniques still target a small number of viral genes for detection. Our patented multi-padlock RCA (MP-RCA) [14], however, can overcome the critical issues of qPCR with primer recognition of SARS-CoV-2 variants and the complicated temperature control system. Instead of utilizing temperature changes to amplify DNA, RCA utilizes enzymes which exclusively recognize circular DNA segments to specifically amplify target sequences [15]. Therefore, RCA is an isothermal enzymatic process that linearly amplifies a single molecule of target DNA or RNA into a long single-stranded DNA product, detectable by fluorescent or colorimetric approaches [16,17,18,19,20,21]. The potential of RCA in diagnostic testing has not been fully explored, particularly in applications such as viral detection and diagnostics. We previously developed an RCA-based bioassay for the fluorescent detection of DNA and proteins, demonstrating high sensitivity and a low limit of detections, but have not explored this method for viral detection [22,23]. Recently, RCA has been utilized to enhance immunofluorescence sensitivity to visualize SARS-CoV-2 localization in human tissue samples [24]. In this study, padlocks specific for SARS-CoV-2 RNA were able to bind and amplify the signal adequately to identify the localization of virus. Additionally, other researchers have applied RCA for viral detection such as for influenza virus. However, this assay could not reach the desired sensitivity due to the weak signal produced by its linear amplification method [25,26]. This could be addressed using MP-RCA, as combining multiple padlocks with simultaneous signal amplification will enhance the sensitivity.

Utilizing the multi-padlock approach to RCA, the number of targeted genetic regions of interest and their sensitivity can both be improved [27]. Therefore, this approach is well suited for the diagnosis of a rapidly mutating virus such as SARS-CoV-2. A variant with multiple mutations in protein-coding genes, or even entire gene-dropouts such as S-gene target failure (SGTF), could still be positively identified due to the full-genome targeting of padlocks [28]. Additionally, the simplicity of the MP-RCA reaction can reduce the time required for amplification and detection, as the reaction can be performed directly on a plate reader without the reverse-transcription steps required for PCR methods. The potential versatility of the output is another major appeal of MP-RCA, including the ability to generate colorimetric output not requiring fluorescent readers [16,17,18]. 

In this study, we have developed a fast and simple viral RNA detection method. The multi-region padlock hybridization approach provides better sensitivity to single-padlock RCA linearly corresponding to the number of multi-regions hybridizations, maintaining this sensitivity even with several mutations in the target RNA fragment. Our method increases the specificity and sensitivity of the traditional RCA by targeting multiple regions of the different coding genes of SARS-CoV-2, including the open reading frame (ORF)-, nucleocapsid (N)-, and spike (S)-, RNA-dependent RNA polymerase (RdRP)-, and envelop (E)-protein. We analyzed 55 samples that were determined positive through qPCR and were able to detect the SARS-CoV-2 virus in 45 of these via this RCA method with multi-region hybridizations. Additionally, using 40 different padlocks against the SARS-CoV-2 sequence allowed the detection of the virus even in SGTF variants. This ability to detect mutated variants decreases the risk of false negatives, while the linear amplification method of RCA provides a reduced chance of false positives. Minimizing false positive or negative results is essential to avoid exacerbating the public health crisis [1,29,30]. This assay proved easy to perform and only required a plate reader equipped with incubation. In future studies, we will expand this platform to test additional viruses such as influenza and implement an on-chip assay for further simplified experimentation and analysis.

## 2. Materials and Methods

### 2.1. Reagents

Synthetic DNA oligomers and the positive control RNA fragments were purchased from Integrated DNA Technologies (IDT, Coralville, IA, USA) for the padlock probes (Supplementary Table S1), circular DNA templates (Supplementary Table S2), and the RCA primer (CATTGCTGCTGGCTG). The polymerase enzyme phi29 and the phi29 reaction buffer were both purchased from the Monserate Biotech group (San Diego, CA, USA). SplintR ligase enzyme was used for the ligation of the padlock probe hybridized with the target RNA sequence and purchased from New England Biolabs (NEB, Ipswich, MA, USA). For the RCA reaction, various supplement chemicals were required as below. ATP (#-P0756S), dNTP, and BSA were purchased from NEB. MnCl2 was purchased from Sigma-Aldrich (Saint Louis, MO, USA). For the quantification of the DNA fragments from the RCA reaction, SYBR gold was used and purchased from Invitrogen (ThermoFisher Scientific, Waltham, MA, USA).

### 2.2. RNA Isolation and Preparation

This work was carried out at the Northeastern University COVID-19 response laboratory, at the Life Sciences Testing Center (LSTC; Burlington, MA, USA), which was established for routine testing for university affiliates. Polyester nasal swabs are collected in empty 3 mL BD Vacutainer tubes (Becton, Dickinson and Co., Franklin Lakes, NJ, USA) and resuspended in 3 mL of viral transport media (VTM; Redoxica, Little Rock, AR, USA), then stored at −80 °C. For RNA isolation, samples are thawed and the Thermo MagMAX protocol was followed using Agilent liquid handler (Agilent Technologies, Santa Clara, CA, USA). Briefly, 200 µL of sample volume was used along with 275 µL of lysis buffer and MagMAX RNA binding beads (ThermoFisher). We then added 10 µL of Proteinase K and 10 µL of MS2 phage extraction control into the above mix in a 1 mL 96-well plate. The plate was vortexed for 2 min followed by incubation for 5 min at 65 °C. Then, the plate was vortexed again for 5 min and incubated at room temperature on a magnetic stand for RNA-bound bead separation. Beads were washed three times with the wash buffer followed by one wash with 80% ethanol. The final elution was performed in 50 µL of elution buffer. Real-time qPCR was performed with the ThermoFisher TaqPath COVID-19 combo kit on Applied Biosystems Fast 7500 DX qPCR machines using 10 µL of eluent from the RNA extraction. Similarly, 10 µL was used for the MP-RCA diagnostic method. Positive and negative SARS-CoV-2 samples were determined by the LSTC-based FDA-approved guidelines for the TaqPath COVID-19 assay. Patient samples were de-identified by the LSTC prior to use for this study, and as such, the study was not deemed as research on human subjects by Northeastern University’s IRB.

### 2.3. Padlock Design

**Figure 1:**
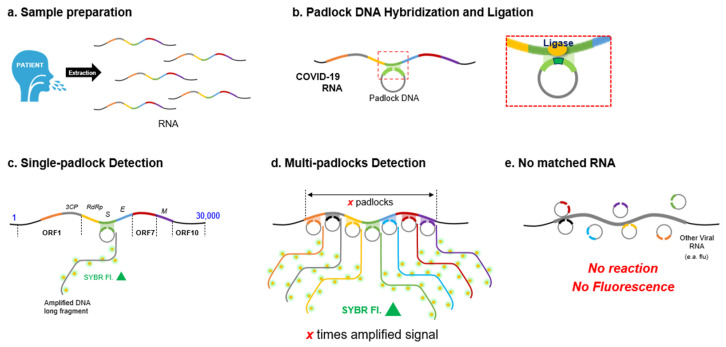
Graphical overview of MP-RCA diagnostic approach. (**a**) RNA is purified from patient samples. (**b**) DNA padlocks recognize specific target sequences in SARS-CoV-2, anneal, and are circularized via ligase. (**c**) Circularized padlocks are amplified via phi29, and the amplified DNA product is labeled in real time with SYBR gold or other fluorescent DNA stains. (**d**) Recognition of multiple padlocks to different regions of the SARS-CoV-2 genome increases amplification and thus fluorescent signal intensity. (**e**) In the absence of target sequences of RNA, no amplification will occur.

The multi-region hybridization padlocks (multi-padlocks) were designed using the SARS-CoV-2 genome (NC_045512), as shown in Figure 1. The multi-region padlocks with the complementary sequence to the reference genome are hybridized using both left and right arms for the ligation reaction after annealing with target RNA. One arm of each padlock possesses a phosphate functional group at the 5′ end to ligate the circular immediate DNA. Each padlock in this work was designed to bind the different region of the target RNA genome. Thus, the right arms with the 5′-phosphate group were reversed to the target SARS-CoV-2 genome, while the left arms with the 3′-end were complementary sequenced to the target. The middle region of each padlock also contains a target sequence complementary to the RCA initiation primer binding to lead the RCA reaction using phi29 polymerase.

### 2.4. MP-RCA Reaction System and Fluorescence Measurement

For the RCA reaction, 50 µL of the reaction mixture was prepared and designed to achieve the final concentrations of 1X Monserate RCA reaction buffer, 1 mM of dNTPs, 0.25 mM of ATP, 2 mM of MnCl_2_, 0.05 mg/mL of BSA, diluted SYBR gold (1:1000 dilution), 10 nM of RCA primer, 2.5 U of phi29 polymerase, 2.5 U of SplintR ligase, and 100 pM of padlocks. Every RCA reaction included padlocks with a final concentration at 100 pM, irrespective of the number of different padlocks present in the RCA reaction.

To acquire better efficiency of the RCA reaction, RNA denaturation and padlock annealing were applied by heating the microplate containing the RCA mixture at 45 °C for 15 min. Then, the RCA mixture was incubated at 37 °C for 3 h in the microplate reader (Varioskan LUX multi-mode microplate reader, ThermoFisher Scientific). Because SYBR gold was used to quantify the long DNA fragment from the 3 h of RCA reaction, the emission was measured at 495 nm with excitation at 537 nm. Ligated padlocks undergo continuous amplification, creating a linear increase in fluorescent signal that reduces over time.

We utilized 3 variations of negative controls for these experiments: the RCA reaction enzyme mixture alone, reaction mix with padlocks but no RNA sample included, and reaction mix with RNA but no padlocks included. A positive result was determined by a statistically significant increase in fluorescent signal from our padlock-only negative control based on a Student t-test for single comparisons and ANOVA for multiple comparisons. A negative result was in turn a statistically insignificant change in fluorescence compared to the negative control.

### 2.5. Circularization of Positive Control for MP-RCA Reaction

As a successful ligated padlock determines proper RNA detection, we utilized pre-ligated padlocks as positive controls for all reactions. The reaction contained 1X SplintR ligase buffer, 1X NEB buffer, 1X exonuclease I buffer, 20 µM of the target synthetized RNA template (IDT), 2.5 U of SplintR ligase, 3 U of thermostable exonuclease I, 1 U of exonuclease III, and the multi-padlock (4 µM).

Briefly, padlocks were ligated to a synthetic short sequence of target RNA (Custom RNA oligos, IDT) for 1 h at 25 °C. After ligation, ligase was inactivated by heating the reaction mix to 65 °C for 10 min. Subsequently, solutions containing ligated padlocks were treated with a mixture of Exonuclease I and Exonuclease III (NEB) at 37 °C for 30 min to digest any non-ligated padlocks. Exonuclease were inactivated at 80 °C for 10 min. The ligated circles were then purified using Oligo Clean-up and Concentration kits (Norgen BioTEK, Thorold, ON, Canada) as per the manufacturer’s protocol. The resultant ligated padlock solution, which was circularized DNA, was purified and eluted into the final product in a volume of 100 μL.

## 3. Results and Discussion

### 3.1. In Silico Comparison Analysis for the Design of the Padlocks

The padlock sequences were designed to hybridize to target different coding genes of SARS-CoV-2 including the open reading frame (ORF)-, nucleocapsid (N)-, spike (S)-, RNA-dependent RNA polymerase (RdRP)-, and envelop (E)-protein. Since the designed padlocks cover almost the entire SARS-CoV-2 genome, there are concerns about unexpected hybridization to the similar respiratory viral families.

Therefore, in silico computational hybridizations analysis has been applied to avoid an unexpected interference in the hybridization of the padlocks toward other viruses, including similar respiratory virus families. In total, 28 viruses were compared in the model, including influenza A/B, human coronavirus 229E/OC43/HKU1/NL63, Middle East respiratory syndrome-related coronavirus (MERS), and other pathogenic viruses (Supplementary Table S4). This computational analysis was also used to detect any padlocks that might hybridize with each other in the reaction. As a result of the computational synchronization between the arm sequences of the padlocks and the representative respiratory virus families, we excluded padlock designs that may undergo unexpected hybridization and produce false positive/negative results. A total of 199 unique padlock sequences specific for the SARS-CoV-2 genome were designed. This number was narrowed down to 60 padlocks distributed across all major coding regions of the genome, for experimental simplicity. The chosen padlock sequences were experimentally reaffirmed to have synchronized to the target SARS-CoV-2 RNA sequence. However, experimentally, we found some cross-reactivity between padlocks. The cross-reactive padlocks were removed, leaving 40 padlocks for the final reaction mix (Supplementary Table S1).

### 3.2. Recovery and Sensitivity Test of Synthesized RNA for the RCA Using SYBR Gold

We first sought to find the optimal concentration of padlocks for the best signal separation of positive samples from negative controls. Different concentrations of circular DNA templates were tested to evaluate the efficiency of the suggested RCA reaction. First, the self-ligated circular DNA template was synthesized using only padlock #1 (Supplementary Table S2), as described in the Materials and Methods section. Then, the RCA reaction was performed with different concentrations of circular DNA controls (10 nM, 100 pM, 100 fM, and 10 fM). The different concentrations of ligated circular DNA templates were used to determine the limit of detection for this assay. Quantification of the RCA reaction was measured by the fluorescent signal of SYBR gold, a dye that produces a strong fluorescence when conjugated to nucleic acids.

**Figure 2:**
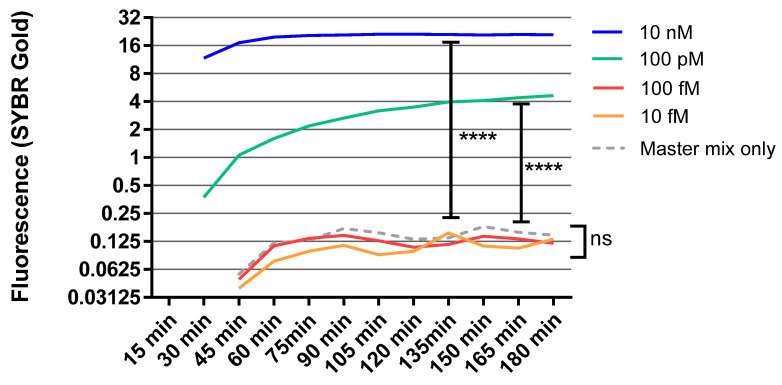
RCA was performed using ligated positive controls in shown concentration with SYBR gold. The detection limit of SYBR gold was 100 pM. Fluorescence data was observed at excitation 495 nM and emission 537 nM for 3 h at 15 min interval. Student *t*-test was performed for statistical comparison. Y axis is in log(2). **** = *p* < 0.0001, ns = *p* > 0.05.

An increase in fluorescent signal was observable with a minimum of 100 pM of the circular DNA template when compared to a negative control that consisted of master mix alone without the circular DNA template (Figure 2). This signal separation further increased with 10 nM of circular DNA template, producing a peak fluorescent signal within only 60 min. This is a significantly shorter time to peak signal strength than the conventional qPCR method. The minimum effective concentration of padlocks (100 pM) was utilized for subsequent experiments to conserve reagent volumes and minimize the signal in negative controls.

### 3.3. MP-RCA Optimization to the Imitated SARS-CoV-2 RNA

**Figure 3:**
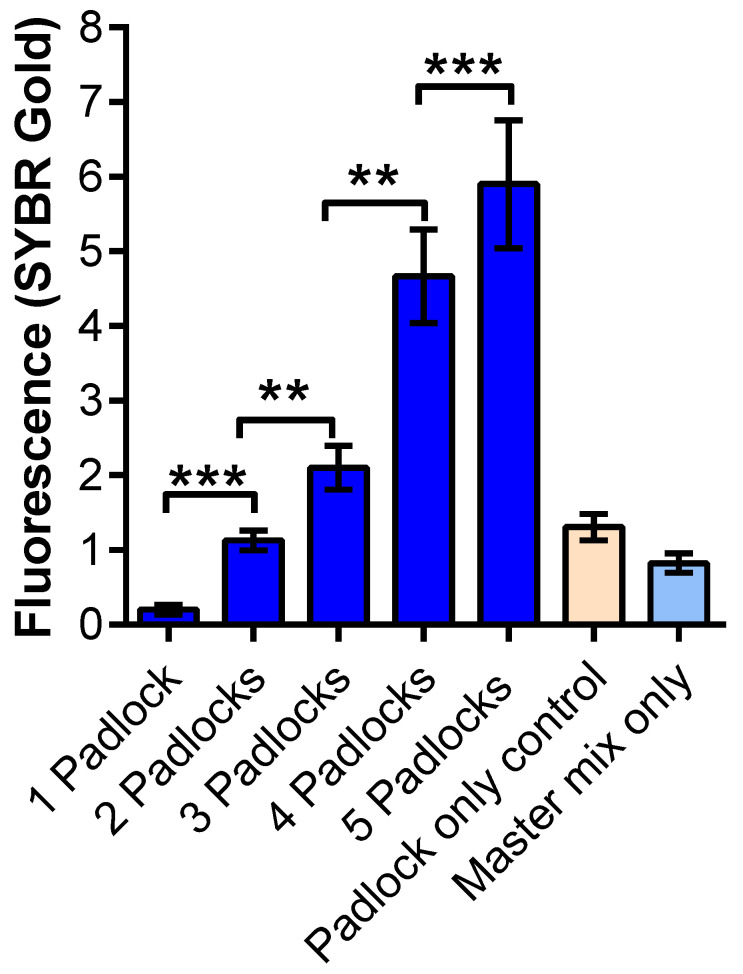
IDT targets A-35, A-36, and A-37 (10 nM) were used with different padlock mix n 10 nM concentration. Mix 1 (padlock 1), mix 2 (padlocks 1 and 2), mix 3 (padlocks 1–3), mix 4 (padlocks 1–4), and mix 5 (padlocks 1–5). Student *t*-test was performed to compare significance between two combinations of padlocks. ** *p* < 0.005 and ***, *p* < 0.0001.

To optimize the MP-RCA approach, we used synthesized custom RNA fragments (Figure 3 and Supplementary Table S2) as the detection targets. These targets are around 120 bp long sequences of SARS-CoV-2 and are used as positive controls in various diagnostic techniques. To verify whether using multi-padlocks linearly increases the amplification as well as the fluorescence signal from SYBR gold, we used a mix of three targets ranging from one to five padlocks. As can be seen in Figure 3, the fluorescent signal of SYBR gold increased with a linear correlation as the number of padlocks increased, corresponding to the number of target RNA fragments ranging from one to five. This result demonstrates that our multi-padlock approach has advantages in sensitivity over conventional RCA with the use of only a single padlock. Among the negative control samples, the padlock-only sample showed a slight fluorescence signal due to the binding of SYBR gold. The fluorescent intensity of this control was slightly higher than the baseline as this control contained all five padlocks and increasing padlock concentration shows a correlation to increased background noise. The difference between one padlock and the negative controls was not statistically significant (*p* > 0.05).

### 3.4. Diagnostic Test for RCA Detection of SARS-CoV-2 Patient Samples

**Figure 4:**
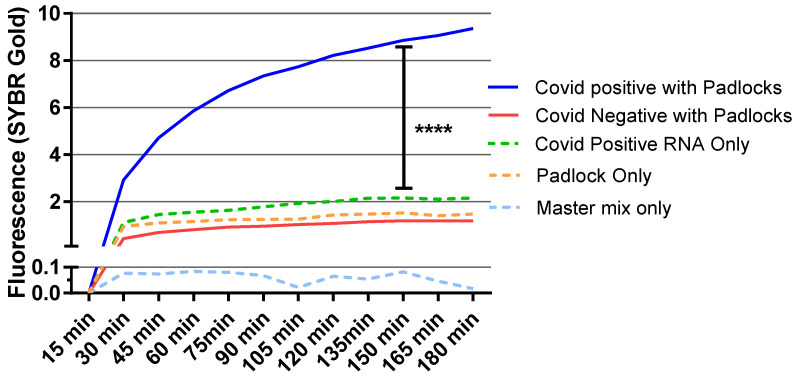
RCA was used to analyze positive and negative samples (averages of 34 positive and 44 negative samples) with SYBR gold. Padlocks were used at 100 pM concentrations. SYBR gold fluorescence was observed for 3 h at 15-min intervals at excitation 495 nm and emission at 537 nm. The statistical significance was calculated using one way analysis of variance (ANOVA) between indicated groups. ****, *p* < 0.0001.

After optimizing RCA with the target synthetic RNA fragments, we applied this MP-RCA approach to COVID patient samples. Nasal swab samples were collected from university employees as a part of routine testing, which had previously been analyzed by qPCR and stored at −80 °C. The target viral RNA from both positive and negative samples was isolated. We used the MP-RCA approach on 95 patient samples, 45 of which tested positive and 50 negative through qPCR. The criterion chosen for a positive result through MP-RCA was a statistically significant increase (*p* < 0.05) in fluorescent signal as compared to a patient-specific negative control with no padlocks present. This method of negative control was chosen to ensure positive signals were not generated directly from patient RNA. From the 45 positive samples, the MP-RCA assay diagnosed 35 patients as positive with a clear high amplification signal, whereas in negative samples, only six samples were detected as false positive (Figure 4). Samples that tested positive through qPCR but not through MP-RCA were deemed negative due to a lack of amplification above the baseline determined from patient samples run in the absence of padlocks (patient-specific negative controls).

While these false positives had higher signals than the negative control samples, they still produced a lower signal than the true-positive samples. Additionally, several of the false-negative samples resulted from above-average signal amplification in the negative controls. With a specific fluorescent intensity cutoff selected for the determination of positive detection rather than statistical significance, we expect false negatives to be reduced, and most false positives to be eliminated from this assay. PCR Ct values and corresponding MP-RCA results are presented in Supplementary Table S3.

### 3.5. Detection of S-Gene Target Failure Variants with Multi-Padlocks

**Figure 5:**
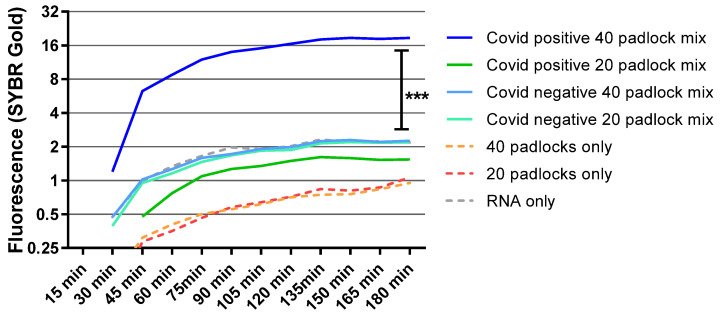
RNA was isolated from previously known S-gene dropout samples (from a total of 10 different patient samples, 5 for each combination), and RCA was performed with either 20 or 40 mix of different combination of multi-padlocks. SYBR gold fluorescence was measured for 3 h at 15-min intervals at excitation 495 nm and emission 537 nm. The statistical significance was calculated using one-way ANOVA between different groups. Y axis is in log(2). ***, *p* < 0.0001.

Next, we sought to apply RCA with SGTF variants of SARS-CoV-2. RNA was isolated from previously confirmed (with qPCR) SGTF samples. We found that increasing the number of padlocks from 20 to 40 enhanced the amplification signal (Figure 5). As expected, this effect was negligible with RNA from SARS-CoV-2-negative samples. We also verified our results using the same sample (sample from one patient divided into two parts) with these two combinations of the mix (Supplementary Figure S1). Overall, our data suggested that MP-RCA can detect many SARS-CoV-2 variants, including any gene-dropouts (Supplementary Table S3 shows CT values of qPCR in samples that have S-genes or SGTF variants).

Combining all of the tested patient samples, we provide evidence that the MP-RCA reaction worked well in 45 out of 55 positive patient samples (81.82% efficiency). We also observed six false positives out of 50 negative samples (88.0% efficiency). Though our current method was less reproducible than qPCR, this MP-RCA was found to be able to detect virus at clinically relevant concentrations, including samples with high quantitative cycle (Cq) values measured by qPCR (Supplementary Table S3). The greatest advantage of this assay is in detecting newly discovered mutant variants. Some conventional qPCR kits may not effectively detect of S-gene target failure (SGTF) variants. The MP-RCA approach proved to be successful in detecting S-gene dropout variants and most other patient samples tested. Therefore, the diagnostic results in this study indicate a potential alternative to detecting all types of emerging variants using the multi-padlock approach. MP-RCA also requires less time for diagnosis, with positive patient samples detectable within 30 to 45 min. Additional amplification techniques, alternative fluorescent detection methods, or simply adding more padlock probes may further improve the sensitivity of this assay [31,32,33].

## 4. Conclusions

This study has established that the MP-RCA approach can be a particularly useful future diagnostic tool to overcome the limitation of other methods in detecting multiple newly emerging variants of SARS-CoV-2, with reduced experimental complexity compared to qPCR. There are also many other potential directions in which MP-RCA may be employed to produce novel diagnostic assays. This method can be readily implemented to test many viruses, as padlocks can be efficiently designed for other genomic sequences. Additionally, MP-RCA may help identify strains of viruses, as this assay can be modified to allow the selective determination of which padlocks (and thus, which regions of the genome) are recognized by the assay [34,35,36] In future studies, we plan to incorporate an on-chip analytical method for even greater simplification of this assay, which we expect to improve the sensitivity. Additionally, we plan to apply this technique to a colorimetric assay, allowing for a sensitive test with the potential for the visual identification of a positive result.

## 5. Patents

The rolling-circle amplification (RCA) for amplification method described in this manuscript has been patented (US patent #US11066697B1).

## Data Availability

The data presented in this study are available on request from the corresponding author. The data are not publicly available due to concerns of patent infringement.

## Supplementary Materials

The following supporting information can be downloaded at: https://www.mdpi.com/article/10.3390/diagnostics12092252/s1, Figure S1: Detection of SARS-CoV-2 S-gene dropout variants; Table S1: Padlocks for SARS-CoV-2 genome; Table S2: Synthetic IDT RNA targets and corresponding DNA padlocks; Table S3: Ct Values—RT-qPCR positive samples and MP-RCA results; Table S4: Padlock Specificity Computational Analysis.
