# Supplementary material for: A Novel Rolling Circle Amplification-Based Detection of SARS-CoV-2 with Multi-Region Padlock Hybridization
Source: Diagnostics (Basel). 2022 Sep 18;12(9):2252. doi: 10.3390/diagnostics12092252 (PMC9497765; doi:10.3390/diagnostics12092252)
Supplement: Supplementary file 1 [file diagnostics-12-02252-s001.zip › diagnostics-1907252-supplementary.pdf]

Supplemental Table S1 - Padlocks for SARS-CoV-2 genome		
**S.N.**	**Name**	**Padlock Sequence**
1	20650-S1	/5Phos/GGTAATTGAGTTCTG cag cca gca gca atg aa ccc aac ccg ccc tac ccc cca acc cgc cct acc c t TTAGTGTATGCAGGG
2	20800-S2	/5Phos/CCATTGGTCCCAGAG cag cca gca gca atg aa ccc aac ccg ccc tac ccc cca acc cgc cct acc c t TCAAACCTCTTAGTA
3	21100-S3	/5Phos/AAAGGCTGAGAGACA cag cca gca gca atg aa ccc aac ccg ccc tac ccc cca acc cgc cct acc c t TCAAGGTCCATAAGA
4	21250-S4	/5Phos/GGTTCTAAAGCCGAA cag cca gca gca atg aa ccc aac ccg ccc tac ccc cca acc cgc cct acc c t GGCAAATCTACCAAT
5	21400-S5	/5Phos/AAAGTCCTAGGTTGA cag cca gca gca atg aa ccc aac ccg ccc tac ccc cca acc cgc cct acc c t TTATATTTTAATAGA
6	22450-S11	/5Phos/GTTGTTGACATGTTC cag cca gca gca atg aa ccc aac ccg ccc tac cct gca atg aa ccc aac ccg ccc tac cct GTCACACTCATATGA
7	22900-S12	/5Phos/ATCTGGTAATATTTG cag cca gca gca atg aa ccc aac ccg ccc tac cct gca atg aa ccc aac ccg ccc tac cct GCTTGGTTTTGATGG
8	23050-S13	/5Phos/AAGGCCGTTAAACTT cag cca gca gca atg aa ccc aac ccg ccc tac cct gca atg aa ccc aac ccg ccc tac cct AGGTGGCAAAACAGT
9	23200-S14	/5Phos/ACCATTAAACCTATA cag cca gca gca atg aa ccc aac ccg ccc tac cct gca atg aa ccc aac ccg ccc tac cct CTGTGTAACTCCAAT
10	23500-S15	/5Phos/CACATATGTCTGCAA cag cca gca gca atg aa ccc aac ccg ccc tac cct gca atg aa ccc aac ccg ccc tac cct AATTAATTGTTGAGT
11	B_21_RdRpA1a	/5Phos/ TCACA TTTAG GATAA cag cca gca gca atg aa ccc aac ccg ccc tac ccc cca acc cgc cct acc c t TTAGG CATGG CTCTA
12	B_22_RdRpA1b	/5Phos/ AAGAA CAAGT GGGCC cag cca gca gca atg aa ccc aac ccg ccc tac ccc cca acc cgc cct acc c t TGTAT GTTTG CGAGC
13	B_23_RdRpA1c	/5Phos/ ATAGA AACGG TGTGA cag cca gca gca atg aa ccc aac ccg ccc tac ccc cca acc cgc cct acc c t CTCAT TAGCT AATCT
14	B_24_RdRpA2a	/5Phos/ CATGA CCATT TCACT cag cca gca gca atg aa ccc aac ccg ccc tac ccc cca acc cgc cct acc c t TAGTG AACCG CCACA
15	B_25_RdRpA2b	/5Phos/ TCCTG ATGAG GTTCC cag cca gca gca atg aa ccc aac ccg ccc tac ccc cca acc cgc cct acc c t AGCAG TTGTG GCATC
16	B_28_RdRpA3b	/5Phos/ TTCTA TAAGA CACTC cag cca gca gca atg aa ccc aac ccg ccc tac ccc cca acc cgc cct acc c t TGTCA ACATC TCTAT
17	B_29_RdRpA3c	/5Phos/ AATAT GCGTA AAACT cag cca gca gca atg aa ccc aac ccg ccc tac ccc cca acc cgc cct acc c t AGAAA TGTTT ACGCA
18	B_30_RdRpA4	/5Phos/ ACAAC AGCAT CGTCA cag cca gca gca atg aa ccc aac ccg ccc tac ccc cca acc cgc cct acc c t GTGCT ATTGA AACAC
19	A_56_RdRp2a	/5Phos/ GACAC CAGCTACGGT cag cca gca gca atg aa ccc aac ccg ccc tac ccc cca acc cgc cct acc c t AGTACTACAG ATAGA
20	A_57_RdRp2b	/5Phos/ TTCAA TAATTTTTGA cag cca gca gca atg aa ccc aac ccg ccc tac ccc cca acc cgc cct acc c t GTGGCGGCTA TTGAT
21	A_58_RdRp3a	/5Phos/ CTATA AACAGTTTTT cag cca gca gca atg aa ccc aac ccg ccc tac ccc cca acc cgc cct acc c t GGGTTTTCTA CATCA
22	A_59_RdRp3b	/5Phos/ GCGCA GTAAGGATGG cag cca gca gca atg aa ccc aac ccg ccc tac ccc cca acc cgc cct acc c t ACGCACACAA TCGAA
23	B_32_EA1b	/5Phos/ GATTG TGTGA ATTTG cag cca gca gca atg aa ccc aac ccg ccc tac ccc cca acc cgc cct acc c t TCCGG ATGAA CCGTC
24	B_33_EA1c	/5Phos/ ATAAA TTGGT TCCAT cag cca gca gca atg aa ccc aac ccg ccc tac ccc cca acc cgc cct acc c t CGTCG TCGGT TCATC
25	B_34_EA2a	/5Phos/ AGCTT GTGCT TACAA cag cca gca gca atg aa ccc aac ccg ccc tac ccc cca acc cgc cct acc c t AAGTT CGTAC TCATC
26	B_35_EA2b	/5Phos/ ACCTG TCTCT TCCGA cag cca gca gca atg aa ccc aac ccg ccc tac ccc cca acc cgc cct acc c t ATTAA CTATT AACGT
27	B_36_EA2c	/5Phos/ TACCA CGAAA GCAAG cag cca gca gca atg aa ccc aac ccg ccc tac ccc cca acc cgc cct acc c t TGTAA CTAGC AAGAA
28	B_37_EA3a	/5Phos/ ACACA ATCGA AGCGC cag cca gca gca atg aa ccc aac ccg ccc tac ccc cca acc cgc cct acc c t ATTGC AGCAG TACGC
29	B_39_EA3c	/5Phos/ AACTC TAGAA GAATT cag cca gca gca atg aa ccc aac ccg ccc tac ccc cca acc cgc cct acc c t GACCA GAAGA TCAGG
30	B_50_E	/5Phos/ GCGCA GTAAGGATGG cag cca gca gca atg aa ccc aac ccg ccc tac ccc cca acc cgc cct acc c t ACGCACACAA TCGAA
31	B_01_NA4a	/5Phos/ CTCCC TCAGT TGCAA cag cca gca gca atg aa ccc aac ccg ccc tac ccc cca acc cgc cct acc c t TTGGT GTATT CAAGG

32	B_02_NA4b	/5Phos/ TAGCA GGATT GCGGG cag cca gca gca atg aa ccc aac ccg ccc tac ccc cca acc cgc cct acc c t CGATT GCAGC ATTGT
33	B_03_NA4c	/5Phos/ GCAAT GTTGT TCCTT cag cca gca gca atg aa ccc aac ccg ccc tac ccc cca acc cgc cct acc c t CGTAG AAGCC TTTTG
34	B_04_NA5a	/5Phos/ AGGCT TGACT GCCGC cag cca gca gca atg aa ccc aac ccg ccc tac ccc cca acc cgc cct acc c t ATGAG GAACG AGAAG
35	B_05_NA5b	/5Phos/ TTGAA TTTCT TGAAC cag cca gca gca atg aa ccc aac ccg ccc tac ccc cca acc cgc cct acc c t TACTG CTGCC TGGAG
36	B_14_ORF_A1a	/5Phos/ CAACA CGATG CACCA cag cca gca gca atg aa ccc aac ccg ccc tac ccc cca acc cgc cct acc c t CAACG GCAGT ACAGA
37	B_16_ORF_A1c	/5Phos/ GCACA AGTTG TAGGT cag cca gca gca atg aa ccc aac ccg ccc tac ccc cca acc cgc cct acc c t CCCAC AGGGT CATTA
38	B_17_ORF_A2a	/5Phos/ ACGGT ACAGA CTGTG cag cca gca gca atg aa ccc aac ccg ccc tac ccc cca acc cgc cct acc c t TTCCA CATAC CGCAG
39	B_19_ORF_A2c	/5Phos/ CCGTT TAAAA ACGAT cag cca gca gca atg aa ccc aac ccg ccc tac ccc cca acc cgc cct acc c t ACTTA CACCG CAAAC
40	B_20_ORF_A3a	/5Phos/ GCCTG TGCCG CACGG cag cca gca gca atg aa ccc aac ccg ccc tac ccc cca acc cgc cct acc c t GACAT CAGTA CTAGT

**Supplemental Table S2 - Synthetic IDT RNA targets and corresponding DNA padlocks**	
	**RNA Sequences**
A35	rUrGrA rGrUrU rArUrG rArGrG rArUrC rArArG rArUrG rCrArC rUrUrU rUrCrG rCrArU rArUrA rCrArA rArArC rGrUrA rArUrG rUrCrA rUrCrC rCrUrA rCrUrA rUrArA rCrUrC rArArA rUrGrA rArUrC rUrUrA rArGrU rArUrG rCrCrA rUrUrA rGrUrG rCrArA rArGrA rA
A36	rUrArG rArGrC rUrCrG rCrArC rCrGrU rArGrC rUrGrG rUrGrU rCrUrC rUrArU rCrUrG rUrArG rUrArC rUrArU rGrArC rCrArA rUrArG rArCrA rGrUrU rUrCrA rUrCrA rArArA rArUrU rArUrU rGrArA rArUrC rArArU rArGrC rCrGrC rCrArC rUrArG rArGrG rArGrC rU
A37	rArCrU rGrUrA rGrUrA rArUrU rGrGrA rArCrA rArGrC rArArA rUrUrC rUrArU rGrGrU rGrGrU rUrGrG rCrArC rArArC rArUrG rUrUrA rArArA rArCrU rGrUrU rUrArU rArGrU rGrArU rGrUrA rGrArA rArArC rCrCrU rCrArC rCrUrU
	**Padlocks for IDT targets**
Padlock 1 binds to A35	AGTGC ATCTTGATCC CAGCCAGCAGCAATGAACCCAACCCGCCCTACCCTGCAATGAACCCAACCCGCCCTACCCT TTTGTATATG CGAAA
Padlock 2 binds to A35	GATTC ATTTGAGTTA CAGCCAGCAGCAATGAACCCAACCCGCCCTACCCTGCAATGAACCCAACCCGCCCTACCCT TAATGGCATA CTTAA
Padlock 3 binds to A36	GACAC CAGCTACGGT CAGCCAGCAGCAATGAACCCAACCCGCCCTACCCTGCAATGAACCCAACCCGCCCTACCCT AGTACTACAG ATAGA
Padlock 4 binds to A36	TTCAA TAATTTTTGA CAGCCAGCAGCAATGAACCCAACCCGCCCTACCCTGCAATGAACCCAACCCGCCCTACCCT GTGGCGGCTA TTGAT
Padlock 5 binds to A37	CTATA AACAGTTTTT CAGCCAGCAGCAATGAACCCAACCCGCCCTACCCTGCAATGAACCCAACCCGCCCTACCCT GGGTTTTCTA CATCA

Supplemental Table S3 - CT Values-RT-qPCR Positive Samples and MP-RCA Result					
#	Covid positive samples ID	ORF1ab gene	N gene	S gene	MP-RCA Result
1	2035801464	23.27487	23.61304	23.28181	Positive
2	LWPOAAAA58454b	15.659471	16.66798	15.4466	Positive
3	GBPOAAAA12232a	17.18241	18.77288	17.91845	Positive
4	LWPOAAAA00365B	28.274288	28.44961	29.04769	Positive
5	LWPOAAAA00365A	28.176115	28.30485	28.05944	Positive
6	212522725	10.919122	11.64765	11.40171	Positive
7	212530236	11.386249	12.55814	11.91892	Positive
8	2125300742	17.700424	18.94571	17.46984	Positive
9	2125303035	17.266712	18.80636	17.88756	Positive
10	2125303279	12.567639	13.21923	12.93188	Positive
11	2125303501	16.098845	16.69431	16.51827	Positive
12	2125303808	20.016888	21.33041	20.2876	Positive
13	2123500887	11.829923	12.32806	11.77036	Positive
14	2123600827	17.07823	18.17433	17.03232	Positive
15	2123700466	13.417307	14.48047	13.37219	Positive
16	2123900222	19.242903	19.50288	18.76111	Positive
17	2123900441	17.656605	18.86248	18.36178	Positive
18	2124000029	15.848645	16.24882	15.70552	Positive
19	2124200486	10.159071	21.47816	10.49172	Positive
20	212600098	13.918985	14.60279	14.52399	Positive
21	2125803146	12.557142	12.94538	13.05601	Positive
22	2125903261	18.127544	19.5281	18.26682	Positive
23	2125903489	12.950025	13.59756	14.09471	Positive
24	2126000909	12.502594	14.68838	13.83371	Positive
25	2126001025	15.764762	17.35576	16.83654	Positive
26	2126002199	14.20577	15.51222	14.88552	Positive
27	VWPOAAAA96579	16.380901	16.91995	16.42412	Positive
28	212670698	16.788023	18.31712	17.65634	Positive
29	2125704756	14.285051	15.36371	14.8302	Positive
30	2126402945	16.981188	16.90966	16.5932	Positive
31	2126501550	17.32538	18.70735	17.96566	Positive
32	2126501814	12.683517	14.17823	13.96058	Positive

33	2126503554	10.528878	9.672027	11.04018	Positive
34	2126504366	16.349142	17.58014	16.60923	Positive
35	2126700403	13.782969	15.89645	15.61534	Positive
36	2125300020	24.84858	25.2625	24.27172	Negative
37	2125302959	15.2212105	16.44221	15.78725	Negative
38	2125303232	18.29622	19.73975	18.80582	Negative
39	2123500431	16.560055	17.94736	17.73421	Negative
40	2123500909	11.9387665	15.91224	11.96017	Negative
41	2123600160	13.316713	13.41303	13.44627	Negative
42	21253684	15.521948	17.27747	16.37066	Negative
43	2125901223	12.094004	13.8473	13.37203	Negative
44	2126702976	10.843681	11.82756	11.79091	Negative
45	2126703317	16.764202	17.83091	16.82079	Negative

S-gene Dropout samples CT Values-RT-qPCR and MP-RCA Result

#	Sample Name	ORF1ab Ct	N gene Ct	S gene Ct	MP-RCA Result
1	210604290	16.043627	16.59804		Positive
2	2106303243	23.41151	23.04137		Positive
3	2106304054	21.56134	21.42687		Positive
4	210604290b	16.043627	16.59804		Positive
5	2106400907	18.51223	19.60957		Positive
6	2106500109	18.796625	19.09117		Positive
7	2106705667	15.869253	18.62836		Positive
8	GBPOAAAA13141	24.553534	23.73351		Positive
9	LWPOAAAA02478	30.008303	29.61234		Positive
10	LWPOAAAA26517	29.15502	27.9469		Positive

Supplemental Table S4 – Padlock Specificity Computational Analysis

Padlock Name	Human coronavirus 229E	Human coronavirus OC43	Human coronavirus HKU1	Human coronavirus NL63	Severe acute respiratory syndrome-related coronavirus	Middle East respiratory syndrome-related coronavirus	Adenoviridae	Human metapneumovirus	Human respirovirus 3	Human respirovirus 1
20650-S1	0	0	0	0	≥100	0	0	0	0	0
20800-S2	0	0	0	0	≥100	0	0	0	0	0
21100-S3	0	0	0	0	99	0	0	0	0	0
21250-S4	0	0	0	0	≥100	0	0	0	0	0
21400-S5	0	0	0	0	≥100	0	0	0	0	0
22450-S11	0	0	0	0	≥100	0	0	0	0	0
22900-S12	0	0	0	0	≥100	0	0	0	0	0
23050-S13	0	0	0	0	≥100	0	0	0	0	0
23200-S14	0	0	0	0	≥100	0	0	0	0	0
23500-S15	0	0	0	0	≥100	0	0	0	0	0
B_21_RdRpA1a	0	0	0	0	≥100	0	0	0	0	0
B_22_RdRpA1b	0	0	0	0	≥100	0	0	0	0	0
B_23_RdRpA1c	0	0	0	0	≥100	0	0	0	0	0
B_24_RdRpA2a	0	0	0	0	≥100	0	0	0	0	0
B_25_RdRpA2b	0	0	0	0	≥100	0	0	0	0	0
B_28_RdRpA3b	0	0	0	0	≥100	0	0	0	0	0
B_29_RdRpA3c	0	0	0	0	≥100	0	0	0	0	0
B_30_RdRpA4	0	0	0	0	≥100	0	0	0	0	0
A_56_RdRp2a	0	0	0	0	≥100	0	0	0	0	0
A_57_RdRp2b	0	0	0	0	≥100	0	0	0	0	0
A_58_RdRp3a	0	0	0	0	≥100	0	0	0	0	0
A_59_RdRp3b	0	0	0	0	≥100	0	0	0	0	0
B_32_EA1b	0	0	0	0	≥100	0	0	0	0	0
B_33_EA1c	0	0	0	0	≥100	0	0	0	0	0
B_34_EA2a	0	0	0	0	≥100	0	0	0	0	0
B_35_EA2b	0	0	0	0	≥100	0	0	0	0	0

B_36_EA2c	0	0	0	0	≥100	0	0	0	0	0
B_37_EA3a	0	0	0	0	≥100	0	0	0	0	0
B_39_EA3c	0	0	0	0	≥100	0	0	0	0	0
B_50_E	0	0	0	0	≥100	0	0	0	0	0
B_01_NA4a	0	0	0	0	≥100	0	0	0	0	0
B_02_NA4b	0	0	0	0	≥100	0	0	0	0	0
B_03_NA4c	0	0	0	0	≥100	0	0	0	0	0
B_04_NA5a	0	0	0	0	≥100	0	0	0	0	0
B_05_NA5b	0	0	0	0	≥100	0	0	0	0	0
B_14_ORF_A1a	0	0	0	0	≥100	0	0	0	0	0
B_16_ORF_A1c	0	0	0	0	≥100	0	0	0	0	0
B_17_ORF_A2a	0	0	0	0	≥100	0	0	0	0	0
B_19_ORF_A2c	0	0	0	0	≥100	0	0	0	0	0

**Padlock Name**	**Human orthorubulavirus 2**	**Human orthorubulavirus 4**	**Influenza A virus**	**Influenza B virus**	**Enterovirus**	**Respiratory syncytial virus**	**Chlamydia pneumoniae**	**Haemophilus influenzae**	**Legionella pneumophila**	**Myco-bacterium tuberculosis**
20650-S1	0	0	0	0	0	0	0	0	0	0
20800-S2	0	0	0	0	0	0	0	0	0	0
21100-S3	0	0	0	0	0	0	0	0	0	0
21250-S4	0	0	0	0	0	0	0	0	0	0
21400-S5	0	0	0	0	0	0	0	0	0	0
22450-S11	0	0	0	0	0	0	0	0	0	0
22900-S12	0	0	0	0	0	0	0	0	0	0
23050-S13	0	0	0	0	0	0	0	0	0	0
23200-S14	0	0	0	0	0	0	0	0	0	0
23500-S15	0	0	0	0	0	0	0	0	0	0
B_21_RdRpA1a	0	0	0	0	0	0	0	0	0	0
B_22_RdRpA1b	0	0	0	0	0	0	0	0	0	0
B_23_RdRpA1c	0	0	0	0	0	0	0	0	0	0
B_24_RdRpA2a	0	0	0	0	0	0	0	0	0	0
B_25_RdRpA2b	0	0	0	0	0	0	0	0	0	0
B_28_RdRpA3b	0	0	0	0	0	0	0	0	0	0

B_29_RdRpA3c	0	0	0	0	0	0	0	0	0	0
B_30_RdRpA4	0	0	0	0	0	0	0	0	0	0
A_56_RdRp2a	0	0	0	0	0	0	0	0	0	0
A_57_RdRp2b	0	0	0	0	0	0	0	0	0	0
A_58_RdRp3a	0	0	0	0	0	0	0	0	0	0
A_59_RdRp3b	0	0	0	0	0	0	0	0	0	0
B_32_EA1b	0	0	0	0	0	0	0	0	0	0
B_33_EA1c	0	0	0	0	0	0	0	0	0	0
B_34_EA2a	0	0	0	0	0	0	0	0	0	0
B_35_EA2b	0	0	0	0	0	0	0	0	0	0
B_36_EA2c	0	0	0	0	0	0	0	0	0	0
B_37_EA3a	0	0	0	0	0	0	0	0	0	0
B_39_EA3c	0	0	0	0	0	0	0	0	0	0
B_50_E	0	0	0	0	0	0	0	0	0	0
B_01_NA4a	0	0	0	0	0	0	0	0	0	0
B_02_NA4b	0	0	0	0	0	0	0	0	0	0
B_03_NA4c	0	0	0	0	0	0	0	0	0	0
B_04_NA5a	0	0	0	0	0	0	0	0	0	0
B_05_NA5b	0	0	0	0	0	0	0	0	0	0
B_14_ORF_A1a	0	0	0	0	0	0	0	0	0	0
B_16_ORF_A1c	0	0	0	0	0	0	0	0	0	0
B_17_ORF_A2a	0	0	0	0	0	0	0	0	0	0
B_19_ORF_A2c	0	0	0	0	0	0	0	0	0	0

**Padlock Name**	**Streptococcus pneumoniae**	**Streptococcus pyogenes**	**Bordetella pertussis**	**Mycoplasma pneumoniae**	**Pneumocystis jirovecii**	**Candida albicans**	**Pseudomonas aeruginosa**	**Staphylococcus epidermidis**	**Streptococcus salivarius**
20650-S1	0	0	0	0	0	0	0	0	0
20800-S2	0	0	0	0	0	0	0	0	0
21100-S3	0	0	0	0	0	0	0	0	0
21250-S4	0	0	0	0	0	0	0	0	0
21400-S5	0	0	0	0	0	0	0	0	0
22450-S11	0	0	0	0	0	0	0	0	0

22900-S12	0	0	0	0	0	0	0	0	0
23050-S13	0	0	0	0	0	0	0	0	0
23200-S14	0	0	0	0	0	0	0	0	0
23500-S15	0	0	0	0	0	0	0	0	0
B_21_RdRpA1a	0	0	0	0	0	0	0	0	0
B_22_RdRpA1b	0	0	0	0	0	0	0	0	0
B_23_RdRpA1c	0	0	0	0	0	0	0	0	0
B_24_RdRpA2a	0	0	0	0	0	0	0	0	0
B_25_RdRpA2b	0	0	0	0	0	0	0	0	0
B_28_RdRpA3b	0	0	0	0	0	0	0	0	0
B_29_RdRpA3c	0	0	0	0	0	0	0	0	0
B_30_RdRpA4	0	0	0	0	0	0	0	0	0
A_56_RdRp2a	0	0	0	0	0	0	0	0	0
A_57_RdRp2b	0	0	0	0	0	0	0	0	0
A_58_RdRp3a	0	0	0	0	0	0	0	0	0
A_59_RdRp3b	0	0	0	0	0	0	0	0	0
B_32_EA1b	0	0	0	0	0	0	0	0	0
B_33_EA1c	0	0	0	0	0	0	0	0	0
B_34_EA2a	0	0	0	0	0	0	0	0	0
B_35_EA2b	0	0	0	0	0	0	0	0	0
B_36_EA2c	0	0	0	0	0	0	0	0	0
B_37_EA3a	0	0	0	0	0	0	0	0	0
B_39_EA3c	0	0	0	0	0	0	0	0	0
B_50_E	0	0	0	0	0	0	0	0	0
B_01_NA4a	0	0	0	0	0	0	0	0	0
B_02_NA4b	0	0	0	0	0	0	0	0	0
B_03_NA4c	0	0	0	0	0	0	0	0	0
B_04_NA5a	0	0	0	0	0	0	0	0	0
B_05_NA5b	0	0	0	0	0	0	0	0	0
B_14_ORF_A1a	0	0	0	0	0	0	0	0	0
B_16_ORF_A1c	0	0	0	0	0	0	0	0	0
B_17_ORF_A2a	0	0	0	0	0	0	0	0	0
B_19_ORF_A2c	0	0	0	0	0	0	0	0	0

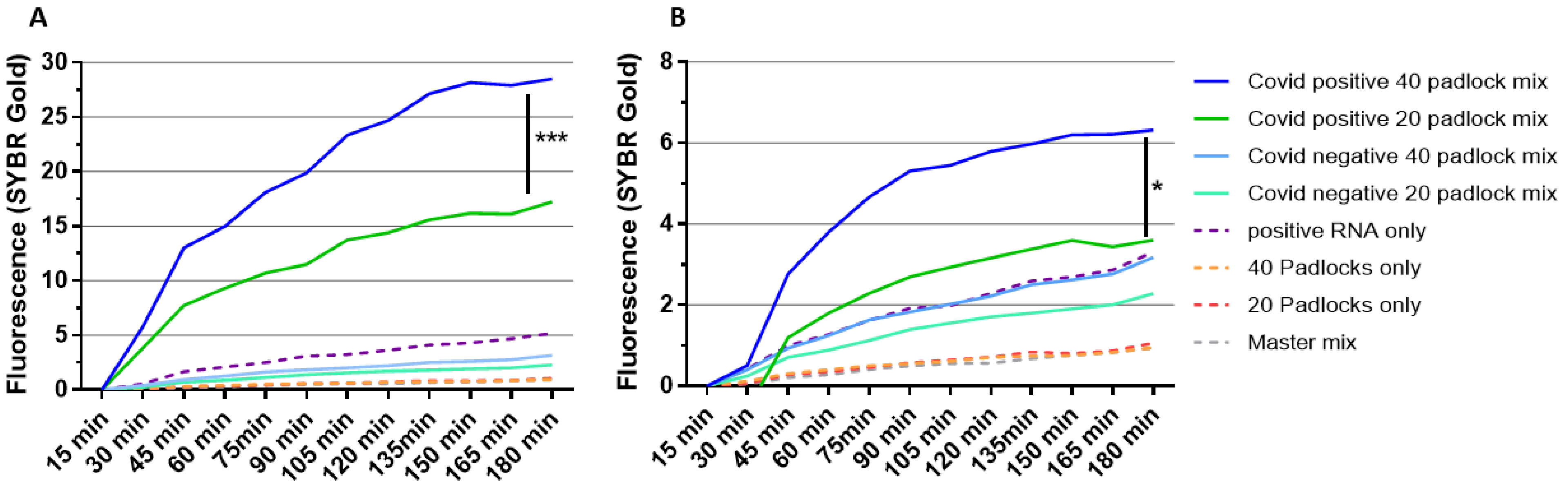

Supplemental Figure S1: Detection of SARS-CoV-2 S-gene dropout variants. RNA was isolated from previously known S-gene dropout sample and RCA was performed with either 20 or 40 mix. A and B are two different positive patient samples having S-gene dropout. SYBR gold fluorescence was measured for 3 hrs at 15 min interval at Excitation 495 nm, Emission 537 nm. The statistical significance was calculated using one way ANOVA between different groups. * P<0.05, *** P<0.0001.
